# Long non-coding RNA tagging and expression manipulation via CRISPR/Cas9-mediated targeted insertion

**DOI:** 10.1007/s13238-017-0464-9

**Published:** 2017-09-05

**Authors:** Tian-Lin Cheng, Zilong Qiu

**Affiliations:** Institute of Neuroscience, State Key Laboratory of Neuroscience, CAS Center for Excellence in Brain Science and Intelligence Technology, Chinese Academy of Sciences, Shanghai, 200031 China

**Dear Editor,**

Long non-coding RNAs (lncRNAs), defined as RNA transcripts longer than 200 nucleotides without the protein-coding ability (Carninci et al., 2005), share many features with protein-coding messenger RNAs (mRNAs) such as polyadenylated 5′ ends and multi-exonic structures (Guttman et al., 2010). Though expression levels are less abundant, lncRNAs outnumber mRNAs with more diverse regulatory functions (Quinn and Chang, 2016). They may serve as decoys, sponges, signals or scaffolds in regulating chromatin conformation, nuclear organization, gene expression, and protein activity in *cis* or *trans* manner (Ulitsky and Bartel, 2013; Quinn and Chang, 2016). LncRNAs are involved in various physiological processes and their loss- or gain-of-function mutations have been implicated in the pathogenesis of human diseases (Wapinski and Chang, 2011). Although their functions have been investigated extensively, manipulation of lncRNAs is challenging, limiting further in-depth analysis for lncRNAs. Efficient and convenient tagging method could be helpful for effective lncRNAs immunoprecipitation to explore lncRNAs-DNA/RNA/protein interactions (Engreitz et al., 2014; Chu et al., 2015). Another challenge is lncRNAs expression manipulation with high efficiency and specificity: Point mutations or insertions and deletions (Indels) are usually insufficient to block lncRNAs functions completely (Cong et al., 2013; Mali et al., 2013). Deleting the whole lncRNA loci or changing lncRNA expression with either clustered regularly interspaced short palindromic repeats (CRISPR)-associated endonuclease Cas9 system, CRISPR interference (CRISPRi) or CRISPR activation (CRISPRa) system have been developed as alternative approaches (Zhu et al., 2016; Liu et al., 2017). However, many lncRNA loci overlap with protein-coding genes and even share common promoter regions, restricting the applications of available tools. In addition, many lncRNAs are cis-acting factors, so traditional overexpression strategy may not work in such conditions. Recently it is shown that targeted insertion could be achieved with CRISPR/Cas9 system via canonical non-homologous end joining (c-NHEJ) pathway without the need for homologous or microhomologous sequences (Schmid-Burgk et al., 2016; Suzuki et al., 2016), so it is plausible to achieve targeted insertion at different sites with one universal donor vector using CRISPR/Cas9 system. As gene trap system has been well-established to disrupt gene functions with selection markers/tags for subsequent functional analysis (Stanford et al., 2001), we here modified gene trap vectors and used CRISPR/Cas9 to establish a scalable tool entitled CTRL (CRISPR-mediated tagging and regulation of lncRNAs) for lncRNA tagging and expression manipulation in mammalian cells. With this method, we successfully tagged lncRNAs at either 5′ or 3′ end. And lncRNA expression status was either stimulated or inhibited reversibly depending on the targeted insertion site.

CTRL system contains a modified gene trap vector, a plasmid expressing *S. pyogenes* Cas9 (SpCas9) and two sgRNAs driven by two U6 promoters respectively (one genome-targeting sgRNA and another donor plasmid-targeting sgRNA) (defined as Cas9-2sgRNA) for lncRNA tagging and expression manipulation purposes. In principle, modified gene trap vector and Cas9-2sgRNA were transfected simultaneously into 293T cells for donor DNA plasmid linearization and targeted insertion at desired genome locus (Fig. 1A). For targeted insertion near transcriptional termination site, a modified polyA trap vector containing CMV-puromycin selection cassette without polyA signal, a specific sgRNA targeting site and 4× MS2 or 24× MS2 tagging sequences were designed (Fig. S1A and S1B). With targeted insertion near transcriptional termination site, puromycin expression is induced to serve as selection marker for cells containing established targeted insertion. Initially, to determine the applicability of CTRL system for lncRNA tagging and purification, genome-targeting sgRNA inside transcriptional termination site of phosphatase and tensin homolog pseudogene 1 (PTENP1) was designed. Then modified polyA trap vector containing 24× MS2 tags and Cas9-2sgRNA/PTENP1 were transfected into 293T cells. After 48 h, puromycin was added at a final concentration of 2 μg/mL and cells were cultured for another 4 days. Survival cells were further incubated in normal growth medium without puromycin for about 1 week to obtain sufficient cells for subsequent analysis. Established targeted insertion and lncRNA tagging were confirmed at the genomic level and mRNA level with PCR/RT-PCR, respectively (Fig. 1B). For PTENP1 purification, 293T cells with established targeted insertion were transfected with plasmid expressing either EGFP or MS2-EGFP fusion protein and cultured for 4 days. Then RNA-immunoprecipitation (RNA-IP) was performed using GFP-Trap_A beads. We found that PTENP1 transcripts were significantly enriched in cells expressing MS2-EGFP fusion protein as compared with cells expressing EGFP protein (Fig. 1C), confirming the reliability of CTRL system for lncRNA purification. Whether CTRL system could be used to manipulate lncRNA expression was further investigated with designed sgRNAs inside transcriptional termination of six lncRNAs ZEB1 antisense RNA1 (ZEB1-AS1), PTENP1, DICER1 antisense RNA 1 (DICER1-AS1), taurine up-regulated 1 (TUG1), HOX transcript antisense RNA (HOTAIR), and myocardial infarction associated transcript (MIAT). As a small amount of puromycin proteins may be produced from CMV-puromycin selection cassette without polyA signal in cells containing targeted insertion with reverse direction, we picked out 2–5 clones for each lncRNA and examined the ratio of clones containing insertion with correct direction. We found that the ratios of targeted insertion with right direction were at least 50%, with 3/6 lncRNAs showing 100% targeted insertion with right direction (Table S1). Established targeted insertion with right direction was confirmed at the genomic level with PCR (Fig. 1D). Then Sanger sequencing was used to examine detailed sequences surrounding the targeted sites for each lncRNA and revealed that targeted insertion was quite precise, as fusion sites in 11/12 clones are generated as expected (Fig. S2). LncRNA tagging was confirmed at mRNA level with RT-PCR (Fig. 1E). The expression level of targeted lncRNAs in 293T cells was examined using real-time quantitative PCR. We found that expression of all six lncRNAs was upregulated significantly, with at least 2-fold changes in 293T cells with established targeted insertion (Fig. 1F). The observed lncRNA upregulation is consistent with previous work reporting that polyadenylation promoted lncRNA turnover (Beaulieu et al., 2012). Many lncRNAs are involved in gene expression regulation with neighboring genes as targets, so the expression of corresponding neighboring genes (ZEB1 vs. ZEB1-AS1; PTENP1-AS1 vs. PTENP1, DICER1 vs. DICER1-AS1, MORC2 vs. TUG1, HOXC11 vs. HOTAIR) was examined. It was revealed that the expression of ZEB1, PTENP1-AS1, and HOXC11 was induced significantly, while DICER1 and MORC2 changed little in 293T cells with established target insertion (Fig. 1G), which indicated that lncRNAs induced by CRISPR/Cas9-mediated targeted insertion were functional.

**Figure 1:**
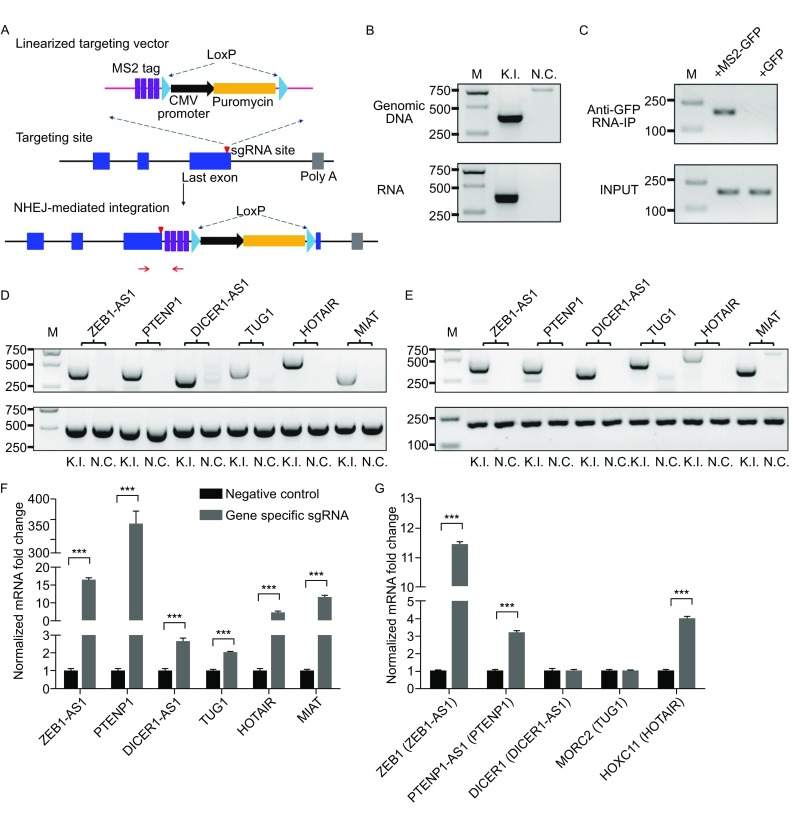
**LncRNA tagging and stimulation by CRISPR/Cas9-mediated targeted insertion inside transcriptional termination**. (A) A scheme for CRISPR/Cas9-mediated targeted insertion inside lncRNA transcriptional termination. Circular modified gene trap vector is initially linearized by vector-targeting Cas9-sgRNA complex. Simultaneously double strand breaks (DSBs) are induced inside transcriptional termination site by genome-targeting Cas9-sgRNA complex. Then linearized vector could be integrated into targeting site via NHEJ mechanism. Red arrow pair shown here represents primers used for PCR confirmation of targeted insertion. (B) DNA agarose gel analysis of targeted insertion for PTENP1 tagging with 24× MS2 tags at both DNA and RNA level with PCR and RT-PCR respectively in 293T cells treated as (A) and cultured for 1–2 weeks after puromycin selection. (C) DNA agarose gel analysis of PTENP1 mRNA purification in 293T cells with RT-PCR. PTENP1 transcripts were purified with GFP-Trap_A beads in 293T cells with established targeted insertion transfected with either MS2-EGFP or EGFP expression vector for 4 days. (D) PCR analysis of targeted integration for six different lncRNAs using genomic DNA of 293T cells with established targeted insertion. (E) RT-PCR analysis of lncRNA tagging with 4× MS2 tags using total RNA of 293T cells with established targeted insertion. (F) Quantitative PCR analysis of targeted lncRNA expression in 293T cells with established targeted insertion. (G) Quantitative PCR analysis of neighboring genes’ expression in 293T cells with established targeted insertion. In (F) and (G), 293T cells without treatment were used as negative control. GAPDH was used as internal control. All experiments were performed independently three times and one representative result was shown. ***P < 0.005 (two-sided Student’s *t*-test)

Targeted insertion inside transcriptional termination site is suitable for lncRNA tagging. However, exogenous fragment insertion inside lncRNA transcripts might disrupt lncRNA functions. Then we designed another six sgRNAs targeting sites after lncRNA transcriptional termination (ZEB1-AS1, PTENP1, DICER1-AS1, TUG1, HOTAIR, and MIAT) and examined the impact of targeted insertions on lncRNA expression in 293T cells (Fig. S3A). Established targeted insertions were confirmed at the genomic level with PCR for all six lncRNAs as described above (Fig. S3B). The expression of all six targeted lncRNAs was also upregulated as described above, though increasing extent was not so dramatic as comparing to cells with targeted insertion inside transcriptional termination site of related lncRNAs (Fig. S3C). The expression changes of neighboring genes were similar as described above, though the upregulation of ZEB1, PTENP1-AS1, and HOXC11 was not so robust as comparing to cells with targeted insertion inside transcriptional termination site of related lncRNAs (Fig. S3D).

Reversion of the established mutation, also referred as rescue experiments, is critical for functional confirmation of targeted genes. Here we added two LoxP sites in the same direction at two ends of linearized donor DNA, so established targeted insertion could be depleted via Cre recombinase delivery (Fig. 2A and 2B). 293T cells with established targeted insertion near transcriptional termination sites of lncRNAs were transfected with Cre expression vector and cultured for about 4 days. Expression changes of targeted lncRNAs and neighboring genes were examined. We found that Cre expression reversed the upregulation of lncRNAs and lncRNA-regulated gene targets in 293T cells with established targeted insertion inside or after transcriptional termination site partially (Fig. 2C–F).

**Figure 2:**
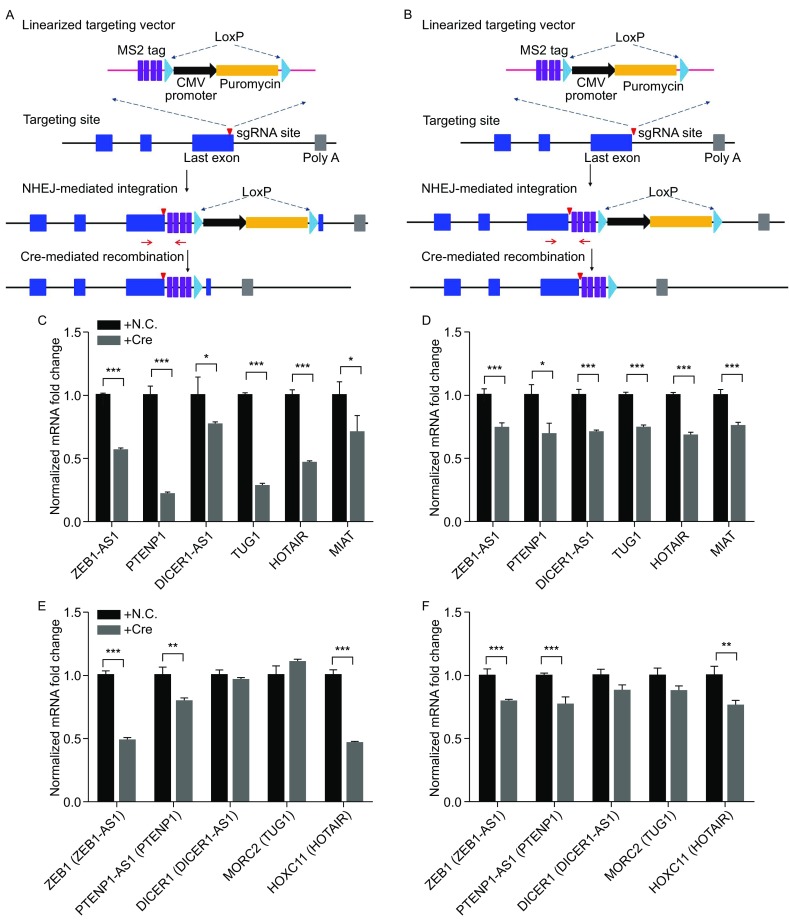
**Reversion of targeted insertion near transcriptional termination by Cre-mediated recombination**. (A and B) A scheme for CRISPR/Cas9-mediated targeted insertion, which is similar to Fig. 1A; Cre-mediated reversion of targeted insertion is as follows: targeted insertion could be deleted with Cre recombinase delivery. (C and D) Quantitative PCR analysis of targeted lncRNA expression in 293T cells with established targeted insertion after transfecting with Cre expression vector. (E and F) Quantitative PCR analysis of neighboring genes’ expression in 293T cells with established targeted insertion after transfecting with Cre expression vector. GAPDH was used as internal control in (C) and (F) analysis. All experiments were performed independently three times and one representative result was shown. *P < 0.05, **P < 0.01, ***P < 0.005 (two-sided Student’s *t*-test)

As targeted insertion near transcriptional termination sites stimulated lncRNA expression, we wondered whether targeted insertion at transcriptional start site would also change lncRNA expression. Here a modified promoter trap vector containing puromycin-polyA cassette without promoter, a specific sgRNA targeting site and 4× MS2 tagging sequences were designed for targeted insertion at transcriptional start site (Fig. S1C). Puromycin-polyA cassette without promoter would be stimulated by endogenous lncRNA promoter in cells with established targeted insertion, which could be depleted by Cre-mediated recombination (Fig. S4A). Six sgRNAs targeting lncRNA transcriptional start sites (ZEB1-AS1, PTENP1, DICER1-AS1, TUG1, HOTAIR, and MIAT) were designed and their impact on lncRNA expression was examined in 293T cells. Established targeted insertions were examined and confirmed at the genomic level with PCR for all six lncRNAs similarly as described above (Fig. S4B). Then we evaluated the corresponding lncRNA expressions in 293T cells containing established targeted insertion and found that the expression of ZEB1-AS1, PTENP1, HOTAIR, and MIAT was stimulated while DICER1-AS1 and TUG1 were inhibited (Fig. S4C), which indicated complicated regulatory effects for targeted insertion at transcriptional start sites. Expression status of neighboring genes was also examined and it was revealed that the expression of ZEB1, PTENP1-AS1, and HOXC11 was upregulated (Fig. S4D), in consistent with the regulatory relationship observed in cells with established targeted insertion near transcriptional termination sites. Cre-mediated reversion of targeted insertion was also evaluated in 293T cells containing established targeted insertion at transcriptional start sites and expected expression reversion was observed. Upregulated lncRNAs including ZEB1-AS1, PTENP1, HOTAIR, and MIAT were inhibited while downregulated lncRNAs including DICER1-AS1 and TUG1 were induced by Cre recombinase (Fig. S4E). LncRNA-regulated target genes including ZEB1, PTENP1-AS1, and HOXC11 were also reversed by Cre recombinase (Fig. 4F), confirming the regulatory relationship between ZEB1/PTENP1-AS1/HOXC11 and ZEB1-AS1/PTENP1/HOTAIR, respectively.

CRISPR/Cas9-mediated targeted insertion has been used for the tagging and correction of protein-coding RNAs (Schmid-Burgk et al., 2016; Suzuki et al., 2016). Here we combined CRISPR/Cas9 with modified gene trap vectors for lncRNA tagging and expression manipulation. Together with the Cre-LoxP system, we further established conditional targeted insertion system for lncRNA expression manipulation for the first time, which was valuable for lncRNA functional analysis. Recently several lncRNA manipulation tools have been established and applied to a large number of lncRNAs (Zhu et al., 2016; Liu et al., 2017). However, as described above, limitations still exist, making it difficult to elucidate the functions of lncRNAs overlapping with other genes and stimulate lncRNA expression specifically (Quinn and Chang, 2016), which could be overcome by our CTRL system. Taken together, our system provides a valuable tool for comprehensive analysis of lncRNA functions and might be used for high-throughput screening in the future.

## Electronic supplementary material

Below is the link to the electronic supplementary material.

Supplementary material 1 (PDF 816 kb)
