# Supplementary material for: Long non-coding RNA tagging and expression manipulation via CRISPR/Cas9-mediated targeted insertion
Source: Protein Cell. 2017 Sep 5;9(9):820–5. doi: 10.1007/s13238-017-0464-9 (PMC6107490; doi:10.1007/s13238-017-0464-9)
Supplement: Supplementary file 1 — Supplementary material 1 (PDF 816 kb) [file 13238_2017_464_MOESM1_ESM.pdf]

Materials and Methods

Construction of donor DNA plasmids

Modified polyA trap vector for targeted insertion near transcriptional termination sites was designed as follows: CMV promoter-puromycin coding sequence-LoxP-donor sgRNA target site-2×MS2 tags -LoxP, was synthesized by Genescript and inserted into vector pUC57-Simple with NdeI and SapI. Here the donor sgRNA site was selected as described previously (Sequence: GGCCAGTACCCAAAAAGCGGGGG)(Schmid-Burgk et al., 2016). 24×MS2 tags were added just after donor sgRNA site to obtain puromycin-selection vector with 24×MS2 tags for RNA-immunoprecipitation (RNA-IP) analysis.

Modified promoter trap vector for targeted insertion near transcriptional start sites was designed as follows: LoxP-donor sgRNA target site- 2×MS2 tags -LoxP-puromycin coding sequence-polyA, was synthesized by Genescript and inserted into vector pUC57-Simple with NdeI and SapI. Donor sgRNA site is the same as modified polyA trap vector.

Construction of MS2-EGFP fusion plasmid

MS2-EGFP fusion plasmid was constructed as follows: the coding sequence of MS2-binding protein was synthesized by Genescript and cloned into EGFP-N2 vector (Clonetech) to obtain MS2-EGFP fusion plasmid.

Construction of dual sgRNA expression vector

42230 vector obtained from Addgene was modified for Cas9 expression and dual sgRNA expression as follows: sgRNA targeting donor sgRNA site was designed,

synthesized, annealed and inserted into 42230 vector via BbsI digestion. Then sgRNA expression cassette (BbsI) was amplified and inserted into 42230-donor sgRNA vector via NotI digestion to obtain 42230-dual sgRNA vector. In subsequent experiments, sgRNA targeting specific genome site was inserted into 42230-dual sgRNA vector via BbsI digestion. All endonucleases were purchased from NEB. Detailed information for all sgRNAs is listed in Table S2.

Cell culture and plasmid transfection

HEK 293T cells were cultured in DMEM medium (Life technologies) containing 10% FBS (Gibco) and 1% Penicillin-Streptomycin antibiotics (Life technologies) in 37℃ incubator with 5% CO2. Cells were plated at a density of 4 ×105 cells/well in a 6-well plate 24 hours prior to plasmid transfection and cultured overnight. Then donor DNA plasmid (1 μg) and 42230-dual sgRNA vector (2 μg) were co-transfected into 293T cells using lipofectamine 2000 reagent (Life Technologies) according to the manufacturer's manuals and cultured for another 48 hours, Then culture medium was replaced with medium supplemented with 2 μg/ml puromycin (Calbiochem) and cultured for another 4 days. Then cells resistant to puromycin were recovered and proliferate to enough number in puromycin-free medium for another 1 week.

For the reversion of targeted insertion with Cre recombinase-induced recombination, puromycin-selected 293T cells were transfected with plasmid expressing either Cre enzyme or EGFP and cultured for another 4 days.

For RNA-IP analysis, 293T cells with targeted insertion were transfected with plasmid expressing either MS2-EGFP fusion protein or EGFP plasmid and cultured for another 4 days.

RNA-IP assay

For RNA-IP assay, 293T cells cultured in 10 cm dish were harvested by trypsinization and resuspended in 1ml freshly-prepared RIPA buffer containing protease inhibitor cocktail (Roche) and RNase inhibitor (Fermentas). Cells were incubated on ice for 20 minutes for complete lysis. 500μl lysis mixture was kept for mock analysis, and another 500μl was used for RNA-IP analysis. Then cell lysis was centrifuged at 16,000×g at 4°C for 10 minutes. Supernatants were collected and added into GFP-Trap_A beads (Chromotek), a GFP-binding protein coupled to agrose beads for immunoprecipitation. After mixed on rotator for 1 hour at 4°C and washed with dilution buffer three times, GFP-Trap_A beads were resuspended with Trizol reagent and total RNA was isolated.

Genomic DNA isolation

Cells were digested in 200 μl lysis buffer containing protein kinase (Millipore) at 50°C overnight and genome DNA was isolated as follows: 200 μl acid-pheno: chloroform (Sangon) was added and mixed thoroughly. After centrifuging for 5 minutes at 12000 rpm. Then supernatant was absorbed and mixed with 400 μl ethanol for DNA precipitation. After centrifuging for 5 minutes at 12000 rpm, supernatant was discarded and washed with 75% ethanol. Then after centrifugation, precipitated

DNA was dissolved with ddH2O for subsequent analysis. Primers used for PCR analysis are listed in Table S2.

RNA purification, reverse transcription and real-time PCR

Total RNA was isolated using Trizol reagent (Invitrogen) according to the manufacturer's manual. Then cDNA was produced from 200 ng to 1 μg purified RNA via reverse transcription using PrimeScript® RT Master Mix (Takara). Quantitative real-time PCR was performed in Rotor-Gene Q machine (Qiagen) using SYBR Green Realtime PCR Master Mix (Toyobo). Experiments were performed in biological triplicates. GAPDH was used as internal control. All qPCR primers are listed in Table S2.

Statistical analysis

Statistical significance based on student's t test analysis was performed with Prism 5.01 (GraphPad Software)

Table S1. Summary of targeted insertion inside transcriptional termination of lncRNAs using CRISPR/Cas9 technology

**targeted lncRNAs**	Clone numer	Right direction	efficiency
**ZEB1-AS1**	2	2	100%
**PTENP1**	2	2	100%
**DICER1-AS1**	2	2	100%
**TUG1**	3	2	66.6%
**HOTAIR**	5	3	60%
**MIAT**	4	2	50%

Table S2. sgRNAs used in this study

sgRNAs targeting N-terminus of lncRNAs	
ZEB1-AS1	GAAAATATTGATACCTGGAC
PTENP1	ATTACTGAGTCATAGGGTAA
DICER1-AS1	GAGCACTGAGAGTGGCGAGA
TUG1	ATGTTTATAAATGCATAGAA
HOTAIR	ACATGTAGCTAAATAGACTC
MIAT	TTCAACAAAGGAGCGTCACT

sgRNAs targeting sites after transcriptional end of lncRNAs	
ZEB1-AS1	TAAATACAGCTAAAGAATAG
PTENP1	ATCGGTGCAGCAAACCAACA
DICER1-AS1	AAGCTCAAAGCGTGAAGATG
TUG1	CAGTGTTGAATAACTTGCCA
HOTAIR	TATGTGAAAGCAACCGACTG
MIAT	AGAAGGGACGTGCTGTTTAG
sgRNAs targeting transcriptional start sites of lncRNAs	
ZEB1-AS1	AACCGAAAGAGAAAAGCGCG
PTENP1	ATGAGAGACAGAGACGGCGG
DICER1-AS1	CCAAAAACTCCGGGCAACTG
TUG1	TTGACCATATTCCACGACCA
HOTAIR	GTCCCAGACCCTGTCAGCCG
MIAT	GGCTGCGGACGAGTGCGGGG

Table S3. qPCR and PCR primers used in this study

ZEB1-AS1 QPCR for	GCCTTCATCCCTTTAGTCCTTAGG
ZEB1-AS1 QPCR rev	GTGAACAGAGTTCATTGTTTAGGG
PTENP1 QPCR for	CGAATAGGTATGTCATCAGAGATC
PTENP1 QPCR rev	CCAGTTTTTTCACATCCTCTCC
DICER1-AS1 QPCR for	GCTTAGCTCGGACAAGGAGATG
DICER1-AS1 QPCR rev	GGTGTCTGTACATCCAGACCA
TUG1 QPCR for	GCTAGAGGTCATGGTCACTG
TUG1 QPCR rev	CTGAACCACACCTTAACCTATCC
HOTAIR QPCR for	CCCCAGCTTGGGACAAAAGTTG
HOTAIR QPCR rev	GGAGGAAGTTCAGGCATTGGG
MIAT QPCR for	GCTTCTCAGTGTGGTTGTGAACG
MIAT QPCR rev	GAGGGGAAAGGGTCACTGTGG
ZEB1 QPCR for	GTGGAAAGCGCTTCTCACAC
ZEB1 QPCR rev	CTCTCTCTCTCGTCCGAGTC
PTENP1-AS1 QPCR for	GTTCATGACTCCTCCTATACC
PTENP1-AS1 QPCR rev	GAAGGGAGAGCTTTATTTCTC
PTEN QPCR for	GTAGAGGAGCCGTCAAATCC
PTEN QPCR rev	CTCTGGATCAGAGTCAGTGG
DICER1 QPCR for	GAGCTTAGGAGATCTGAGGAGG
DICER1 QPCR rev	GTACACCTGCCAGACTGTCTC
MORC2 QPCR for	CTGTGCAATTCCTACCAGAGC
MORC2 QPCR rev	GGTGATGAGGTCCTCAATGTAG
HOXC11 QPCR for	CAAGAAGCGCTGCCCTTATTC
HOXC11 QPCR rev	GCCGGTCTCTGCTCAGTTTC

Primers used for PCR in this study

Universal rev primer:	GGCCGTCGACAGCGTAATCTG
ZEB1-AS1 end for	GCCTTCATCCCTTTAGTCCTTAGG
PTENP1 end for	GCCCTCATGTCCTAATGTGCAG
DICER1-AS1 end for	GCTTAGCTCGGACAAGGAGATG
TUG1 end for	GCTAGAGGTCATGGTCACTG
HOTAIR end for	CCCCAGCTTGGGACAAAAGTTG
MIAT end for	GCTGTGCTGGTGTAAATGGTGAC
ZEB1-AS1 start for	CGTCTCTCCAAACTGGAAAAGGC
PTENP1 start for	CCTCTCAGAAGCTGCAGCCG
DICER1-AS1 start for	CCTGGCGGTGAAAGGTTAATCC
TUG1 start for	GGTTAACAACAGTCCCCTGCTTG
HOTAIR start for	GAAAAACTCTCGCTCCAGTTCCC
MIAT start for	GCTTTCCTGGCCAGGACTCTG

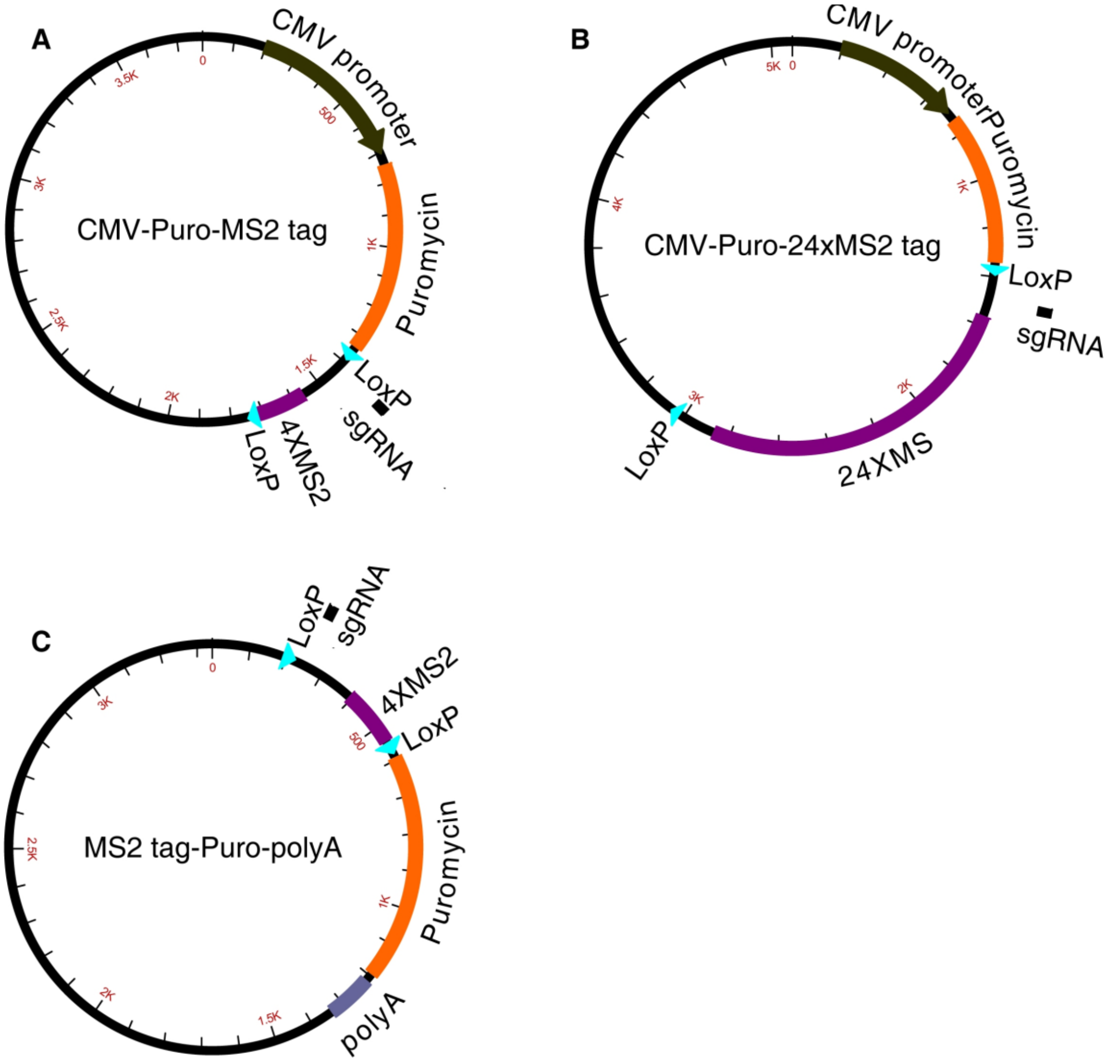

Fig S1. Schematics for Donor DNA plasmids.

(A-B) donor DNA plasmids for targeted insertion near transcriptional termination sites containing either 4×MS2 tags (A) or 24×MS2 tags (B). (C) donor DNA plasmid for targeted insertion near transcriptional start sites containing 4×MS2 tags. Detailed plasmid sequences were provided in appendix.

	LncRNA specific sgRNA sequences<br>**sgRNA** **PAM**	Targeted donor sequence
ZEB1-AS1 sgRNA	GAAAATATTGATACCTGGACTGG	GGCCAGTACCCAAAAAGCGGGGGGGCGCGC
Targeted clone1	GAAAATATTGATACCTG------------	--------------------------------CGGGGGGGCGCGC
Targeted clone2	GAAAATATTGATACCTGG **63bp insertion**	GCGGGGGGGCGCGC
PTENP1 sgRNA	CCCTTACCCTATGACTCAGTAAT	GGCCAGTACCCAAAAAGCGGGGGGGCGCGC
Targeted clone	CCCTTA--------------------------------	--------------------------------CGGGGGGGCGCGC
DICER1-AS1 sgRNA	GAGCACTGAGAGTGGCGAGAAGG	GGCCAGTACCCAAAAAGCGGGGGGGCGCGC
Targeted clone	GAGCACTGAGAGTGGCG------------	--------------------------------CGGGGGGGCGCGC
TUG1 sgRNA	ATGTTTATAAATGCATAGAAAGG	GGCCAGTACCCAAAAAGCGGGGGGGCGCGC
Targeted clone	ATGTTTATAAATGCATA------------	--------------------------------CGGGGGGGCGCGC
HOTAIR sgRNA	ACATGTAGCTAAATAGACTCAGG	GGCCAGTACCCAAAAAGCGGGGGGGCGCGC
Targeted clone	ACATGTAGCTAAATAGA------------	--------------------------------CGGGGGGGCGCGC
MIAT1 sgRNA	TTCAACAAAGGAGCGTCACTTGG	GGCCAGTACCCAAAAAGCGGGGGGGCGCGC
Targeted clone	TTCAACAAAGGAGCGTCA-----------	--------------------------------CGGGGGGGCGCGC

Fig S2. Detailed sequences surrounding fusion sites of six lncRNAs with targeted insertion inside transcriptional termination.

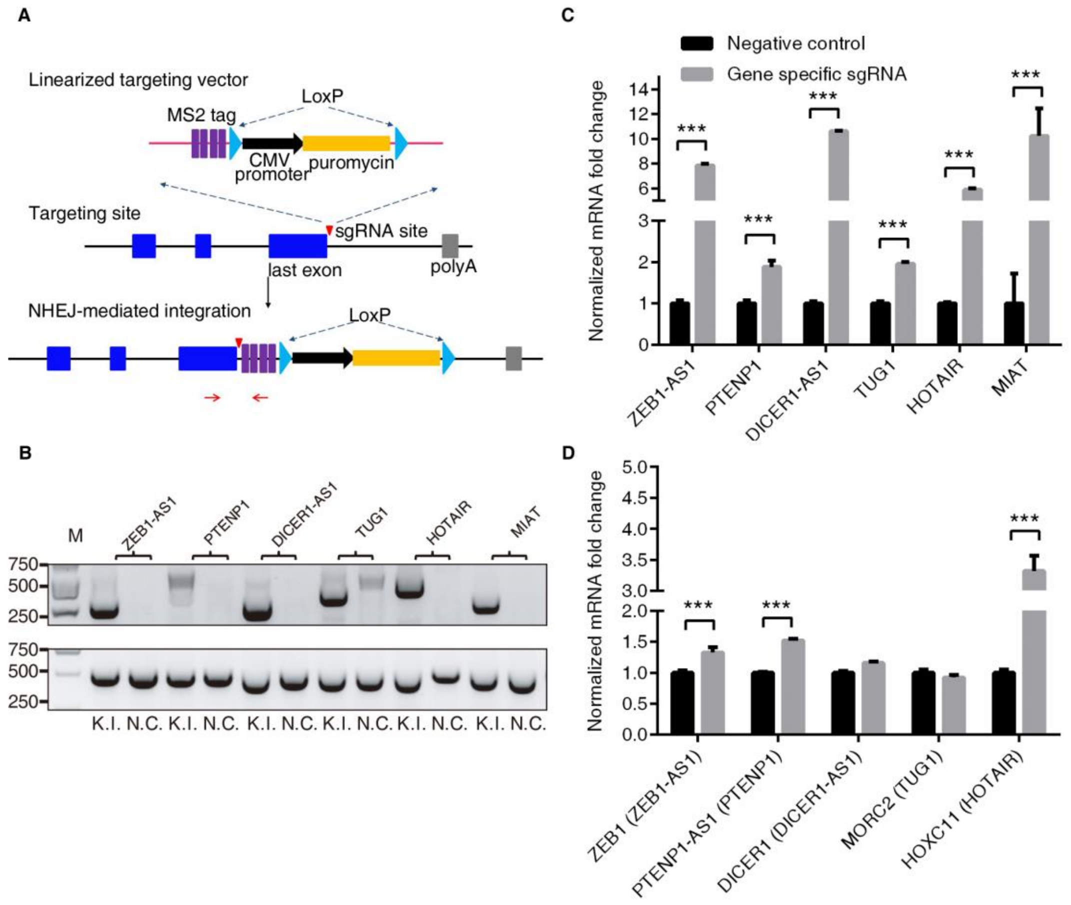

Fig S3. LncRNA stimulation by CRISPR/Cas9-mediated targeted insertion after transcriptional termination.

(A) A scheme for CRISPR/Cas9-mediated targeted insertion after transcriptional termination site similar to Fig. 1A. (B) PCR analysis of targeted integration for six different lncRNAs using genomic DNA of 293T cells with established targeted insertion. (C) Quantitative PCR analysis of targeted lncRNA expression in 293T cells with established targeted insertion. (D) Quantitative PCR analysis of neighboring genes of targeted lncRNA expression in 293T cells with established targeted insertion. 293T cells without treatment were used as negative control and GAPDH was used as internal control in (C) and (D) analysis. All experiments were performed independently three times and one representative result was shown. ***$P<0.005$

(two-sided Student’s *t*-test)

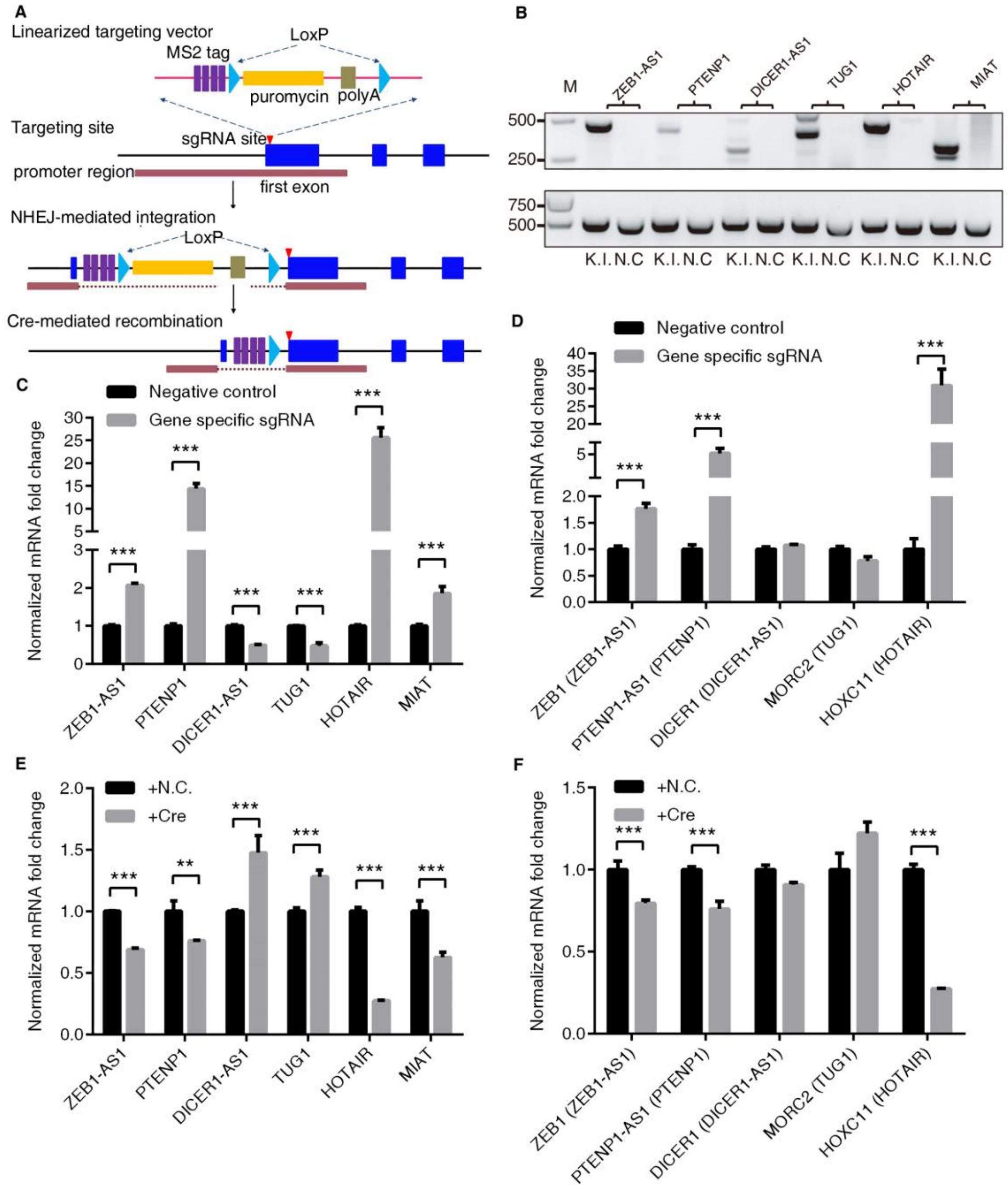

Fig S4. LncRNA manipulation by CRISPR/Cas9-mediated targeted insertion near transcriptional start site.

(A) Schematics of CRISPR/Cas9-mediated targeted insertion near transcriptional start site of lncRNAs. Circular modified gene trap vector is initially linearized by

vector-targeting Cas9-sgRNA complex. Simultaneously double strand breaks (DSBs) are induced inside transcriptional termination site by genome-targeting Cas9-sgRNA complex. After transfecting Cre expression vector, established targeted insertion could be depleted. (B) PCR analysis of targeted integration for six different lncRNAs using genomic DNA of 293T cells with established targeted insertion. (C) Quantitative PCR analysis of targeted lncRNA expression in 293T cells with established targeted insertion. (D) Quantitative PCR analysis of neighboring gene's expression in 293T cells with established targeted insertion. (E) Quantitative PCR analysis of targeted lncRNA expression in 293T cells with established targeted insertion transfected with Cre expression vector. (F) Quantitative PCR analysis of neighboring gene's expression in 293T cells with established targeted insertion transfected with Cre expression vector. GAPDH was used as internal control in (C-F) analysis. All experiments were performed independently three times and one representative result was shown. **$P<0.01$, *$P<0.005$ (two-sided Student's *t*-test)

Appendix

Detailed plasmid sequences and information

CMV-Puro-MS2 tag plasmid

CMV promoter
Puromycin
LoxP
4XMS2
sgRNA

TCGCGCGTTTCGGTGATGACGGTGAAAACCTCTGACACATGCAGCTCCCG
GAGACGGTCACAGCTTGTCTGTAAGCGGATGCCGGGAGCAGACAAGCCC
GTCAGGGCGCGTCAGCGGGTGTTGGCGGGTGTCGGGGCTGGCTTAACTAT
GCGGCATCAGAGCAGATTGTACTGAGAGTGCACCATAATATGGAGTTCCG
CGTTACATAACTTACGGTAAATGGCCCGCCTGGCTGACCGCCCAACGACC
CCCGCCCATTGACGTCAATAATGACGTATGTTCCCATAGTAACGCCAATA
GGGACTTTCCATTGACGTCAATGGGTGGAGTATTTACGGTAAACTGCCCA
CTTGGCAGTACATCAAGTGTATCATATGCCAAGTACGCCCCCTATTGACGT
CAATGACGGTAAATGGCCCGCCTGGCATTATGCCCAGTACATGACCTTAT
GGGACTTTCCTACTTGGCAGTACATCTACGTATTAGTCATCGCTATTACCA
TGGTGATGCGGTTTTGGCAGTACATCAATGGGCGTGGATAGCGGTTTGAC
TCACGGGGATTTCCAAGTCTCCACCCCATTGACGTCAATGGGAGTTTGTTT
TGGCACCAAAATCAACGGGACTTTCCAAAATGTCGTAACAACTCCGCCCC
ATTGACGCAAATGGGCGGTAGGCGTGTACGGTGGGAGGTCTATATAAGCA
GAGCTGGTTTAGTGAACCGTCAGATCCGCTAGGATCCCATGACCGAGTAC
AAGCCCACGGTGCGCCTCGCCACCCGCGACGACGTCCCCAGGGCCGTACG
CACCCTCGCCGCCGCGTTCGCCGACTACCCCGCCACGCGCCACACCGTCG
ATCCGGACCGCCACATCGAGCGGGTCACCGAGCTGCAAGAACTCTTCCTC
ACGCGCGTCGGGCTCGACATCGGCAAGGTGTGGGTCGCGGACGACGGCG
CCGCCGTGGCGGTCTGGACCACGCCGGAGAGCGTCGAAGCGGGGGCGGT
GTTCGCCGAGATCGGCCCGCGCATGGCCGAGTTGAGCGGTTCCCGGCTGG
CCGCGCAGCAACAGATGGAAGGCCTCCTGGCGCCGCACCGGCCCAAGGA
GCCCGCGTGGTTCCTGGCCACCGTCGGAGTCTCGCCCGACCACCAGGGCA
AGGGTCTGGGCAGCGCCGTCGTGCTCCCCGGAGTGGAGGCGGCCGAGCGC
GCCGGGGTGCCCGCCTTCCTGGAAACCTCCGCGCCCCGCAACCTCCCCTTC
TACGAGCGGCTCGGCTTCACCGTCACCGCCGACGTCGAGGTGCCCGAAGG
ACCGCGCACCTGGTGCATGACCCGCAAGCCCGGTGCCTGACAGATCTTAG
ATTATGATAACTTCGTATAGCATACATTATACGAAGTTATTTTACCGGTCG
GGAATTCGGCCAGTACCCAAAAAGCGGGGGGCGCGCCGGGGTCTGGTG
GCAGTGGAGGGGGGTCCTACCCATACGATGTTCCAGATTACGCTTGATTA
CCCCTATGATGTGCCTGACTACGCATGATTACCCATACGATGTTCCAGATT
ACGCTGTCGACGGCCAACATGAGGATCACCCATGTCTGCAGGGCCTTTTT
GGCCAACATGAGGATCACCCATGTCTGCAGGGCCTTTTTGGCCAACATGA
GGATCACCCATGTCTGCAGGGCCTTTTTGGCCAACATGAGGATCACCCAT
GTCTGCAGGGCCGCGCCATAACTTCGTATAGCATACATTATACGAAGTTA
TCGGGATCCATGATATCGCTAGCAAGCTTGCGGCCGCTTCCTCGCTCACTG

ACTCGCTGCGCTCGGTCGTTCGGCTGCGGCGAGCGGTATCAGCTCACTCA
AAGGCGGTAATACGGTTATCCACAGAATCAGGGGATAACGCAGGAAAGA
ACATGTGAGCAAAAGGCCAGCAAAAGGCCAGGAACCGTAAAAAGGCCGC
GTTGCTGGCGTTTTTCCATAGGCTCCGCCCCCCTGACGAGCATCACAAAAA
TCGACGCTCAAGTCAGAGGTGGCGAAACCCGACAGGACTATAAAGATAC
CAGGCGTTTCCCCCTGGAAGCTCCCTCGTGCGCTCTCCTGTTCCGACCCTG
CCGCTTACCGGATACCTGTCCGCCTTTCTCCCTTCGGGAAGCGTGGCGCTT
TCTCATAGCTCACGCTGTAGGTATCTCAGTTCGGTGTAGGTCGTTCGCTCC
AAGCTGGGCTGTGTGCACGAACCCCCCGTTCAGCCCGACCGCTGCGCCTT
ATCCGGTAACTATCGTCTTGAGTCCAACCCGGTAAGACACGACTTATCGC
CACTGGCAGCAGCCACTGGTAACAGGATTAGCAGAGCGAGGTATGTAGG
CGGTGCTACAGAGTTCTTGAAGTGGTGGCCTAACTACGGCTACACTAGAA
GAACAGTATTTGGTATCTGCGCTCTGCTGAAGCCAGTTACCTTCGGAAAA
AGAGTTGGTAGCTCTTGATCCGGCAAACAAACCACCGCTGGTAGCGGTGG
TTTTTTTGTTTGCAAGCAGCAGATTACGCGCAGAAAAAAAGGATCTCAAG
AAGATCCTTTGATCTTTTCTACGGGGTCTGACGCTCAGTGGAACGAAAACT
CACGTTAAGGGATTTTGGTCATGAGATTATCAAAAAGGATCTTCACCTAG
ATCCTTTTAAATTAAAAATGAAGTTTTAAATCAATCTAAAGTATATATGAG
TAAACTTGGTCTGACAGTTACCAATGCTTAATCAGTGAGGCACCTATCTCA
GCGATCTGTCTATTTCGTTCATCCATAGTTGCCTGACTCCCCGTCGTGTAG
ATAACTACGATACGGGAGGGCTTACCATCTGGCCCCAGTGCTGCAATGAT
ACCGCGAGACCCACGCTCACCGGCTCCAGATTTATCAGCAATAAACCAGC
CAGCCGGAAGGGCCGAGCGCAGAAGTGGTCCTGCAACTTTATCCGCCTCC
ATCCAGTCTATTAATTGTTGCCGGGAAGCTAGAGTAAGTAGTTCGCCAGTT
AATAGTTTGCGCAACGTTGTTGCCATTGCTACAGGCATCGTGGTGTCACGC
TCGTCGTTTGGTATGGCTTCATTCAGCTCCGGTTCCCAACGATCAAGGCGA
GTTACATGATCCCCCATGTTGTGCAAAAAAGCGGTTAGCTCCTTCGGTCCT
CCGATCGTTGTCAGAAGTAAGTTGGCCGCAGTGTTATCACTCATGGTTATG
GCAGCACTGCATAATTCTCTTACTGTCATGCCATCCGTAAGATGCTTTTCT
GTGACTGGTGAGTACTCAACCAAGTCATTCTGAGAATAGTGTATGCGGCG
ACCGAGTTGCTCTTGCCCGGCGTCAATACGGGATAATACCGCGCCACATA
GCAGAACTTTAAAAGTGCTCATCATTGGAAAACGTTCTTCGGGGCGAAAA
CTCTCAAGGATCTTACCGCTGTTGAGATCCAGTTCGATGTAACCCACTCGT
GCACCCAACTGATCTTCAGCATCTTTTACTTTCACCAGCGTTTCTGGGTGA
GCAAAAACAGGAAGGCAAAATGCCGCAAAAAAGGGAATAAGGGCGACA
CGGAAATGTTGAATACTCATACTCTTCCTTTTTCAATATTATTGAAGCATTT
ATCAGGGTTATTGTCTCATGAGCGGATACATATTTGAATGTATTTAGAAAA
ATAAACAAATAGGGGTTCCGCGCACATTTCCCCGAAAAGTGCCACCTGAC
GTCTAAGAAACCATTATTATCATGACATTAACCTATAAAAATAGGCGTAT
CACGAGGCCCTTTCGTC

CMV-Puro-24XMS2 tag plasmid

CMV promoter

Puromycin

LoxP
24XMS2
sgRNA
tcgcgcgtttcggtgatgacggtgaaaacctctgacacatgcagctcccggagacggtcacagcttgtctgtaagcggatg
ccgggagcagacaagcccgtcagggcgcgtcagcgggtgttggcgggtgtcggggctggcttaactatgcggcatcag
agcagattgtactgagagtgcaccataatATGGAGTTCCGCGTTACATAACTTACGGTAAAT
GGCCCGCCTGGCTGACCGCCCAACGACCCCCGCCCATTGACGTCAATAAT
GACGTATGTTCCCATAGTAACGCCAATAGGGACTTTCCATTGACGTCAAT
GGGTGGAGTATTTACGGTAAACTGCCCACTTGGCAGTACATCAAGTGTAT
CATATGCCAAGTACGCCCCCTATTGACGTCAATGACGGTAAATGGCCCGC
CTGGCATTATGCCCAGTACATGACCTTATGGGACTTTCCTACTTGGCAGTA
CATCTACGTATTAGTCATCGCTATTACCATGGTGATGCGGTTTTGGCAGTA
CATCAATGGGCGTGGATAGCGGTTTGACTCACGGGGATTTCCAAGTCTCC
ACCCCATTGACGTCAATGGGAGTTTGTTTTGGCACCAAAATCAACGGGAC
TTTCCAAAATGTCGTAACAACTCCGCCCCATTGACGCAAATGGGCGGTAG
GCGTGTACGGTGGGAGGTCTATATAAGCAGAGCTGGTTTAGTGAACCGTC
AGATCCGCTAGGATCCCatgaccgagtacaagcccacggtgcgcctcgccacccgcgacgacgtcccca
gggccgtacgcaccctcgccgccgcgttcgccgactaccccgccacgcgccacaccgtcgatccggaccgccacatcg
agcgggtcaccgagctgcaagaactcttcctcacgcgcgtcgggctcgacatcggcaaggtgtgggtcgcggacgacg
gcgccgccgtggcggtctggaccacgccggagagcgtcgaagcgggggcggtgttcgccgagatcggcccgcgcatg
gccgagttgagcggttcccggctggccgcgcagcaacagatggaaggcctcctggcgccgcaccggcccaaggagcc
cgcgtggttcctggccaccgtcggagtctcgcccgaccaccagggcaagggtctgggcagcgccgtcgtgctccccgg
agtggaggcggccgagcgcgccggggtgcccgccttcctggaaacctccgcgccccgcaacctccccttctacgagcg
gctcggcttcaccgtcaccgccgacgtcgaggtgcccgaaggaccgcgcacctggtgcatgacccgcaagcccggtgc
ctgacagatcttagatTATGATAACTTCGTATAGCATACATTATACGAAGTTATTTTA
CCGGTCGGGAATTCGGCCAGTACCCAAAAAGCGGGGGGCGCGCCGGGG
TCTGGTGGCAGTGGAGGGGGGTCCTACCCATACGATGTTCCAGATTACGC
TTGATTACCCCTATGATGTGCCTGACTACGCATGATTACCCATACGATGTT
CCAGATTACGCTGtcgacactagtTACGGTACTTATTGCCAAGAAAGCACGAGC
ATCAGCCGTGCCTCCAGGTCGAATCTTCAAACGACGACGATCACGCGTCG
CTCCAGTATTCCAGGGTTCATCAGATCCTACGGTACTTATTGCCAAGAAAG
CACGAGCATCAGCCGTGCCTCCAGGTCGAATCTTCAAACGACGACGATCA
CGCGTCGCTCCAGTATTCCAGGGTTCATCAGATCCTACGGTACTTATTGCC
AAGAAAGCACGAGCATCAGCCGTGCCTCCAGGTCGAATCTTCAAACGACG
ACGATCACGCGTCGCTCCAGTATTCCAGGGTTCATCAGATCCTACGGTACT
TATTGCCAAGAAAGCACGAGCATCAGCCGTGCCTCCAGGTCGAATCTTCA
AACGACGACGATCACGCGTCGCTCCAGTATTCCAGGGTTCATCAGATCCT
ACGGTACTTATTGCCAAGAAAGCACGAGCATCAGCCGTGCCTCCAGGTCG
AATCTTCAAACGACGACGATCACGCGTCGCTCCAGTATTCCAGGGTTCAT
CAGATCCTACGGTACTTATTGCCAAGAAAGCACGAGCATCAGCCGTGCCT
CCAGGTCGAATCTTCAAACGACGACGATCACGCGTCGCTCCAGTATTCCA
GGGTTCATCgtcgacactagtTACGGTACTTATTGCCAAGAAAGCACGAGCATCA
GCCGTGCCTCCAGGTCGAATCTTCAAACGACGACGATCACGCGTCGCTCC
AGTATTCCAGGGTTCATCAGATCCTACGGTACTTATTGCCAAGAAAGCAC

GAGCATCAGCCGTGCCTCCAGGTCGAATCTTCAAACGACGACGATCACGC
GTCGCTCCAGTATTCCAGGGTTCATCAGATCCTACGGTACTTATTGCCAAG
AAAGCACGAGCATCAGCCGTGCCTCCAGGTCGAATCTTCAAACGACGACG
ATCACGCGTCGCTCCAGTATTCCAGGGTTCATCAGATCCTACGGTACTTAT
TGCCAAGAAAGCACGAGCATCAGCCGTGCCTCCAGGTCGAATCTTCAAAC
GACGACGATCACGCGTCGCTCCAGTATTCCAGGGTTCATCAGATCCTACG
GTACTTATTGCCAAGAAAGCACGAGCATCAGCCGTGCCTCCAGGTCGAAT
CTTCAAACGACGACGATCACGCGTCGCTCCAGTATTCCAGGGTTCATCAG
ATCCTACGGTACTTATTGCCAAGAAAGCACGAGCATCAGCCGTGCCTCCA
GGTCGAATCTTCAAACGACGACGATCACGCGTCGCTCCAGTATTCCAGGG
TTCATCgTCGACGGCCAACATGAGGATCACCCATGTCTGCAGGGCCTTTTT
GGCCAACATGAGGATCACCCATGTCTGCAGGGCCTTTTTGGCCAACATGA
GGATCACCCATGTCTGCAGGGCCTTTTTGGCCAACATGAGGATCACCCAT
GTCTGCAGGGCCGCGCCATAACTTCGTATAGCATACATTATACGAAGTTA
TCGGGATCCATGATATCGCTAGCAAGCTTGCGGCCgcttcctcgctcactgactcgctgcg
ctcggtcgttcggctgcggcgagcggtatcagctcactcaaaggcggtaatacggttatccacagaatcaggggataacg
caggaaagaacatgtgagcaaaaggccagcaaaaggccaggaaccgtaaaaaggccgcgttgctggcgtttttccatag
gctccgcccccctgacgagcatcacaaaaatcgacgctcaagtcagaggtggcgaaacccgacaggactataaagatac
caggcgtttccccctggaagctccctcgtgcgctctcctgttccgaccctgccgcttaccggatacctgtccgcctttctccct
tcgggaagcgtggcgctttctcatagctcacgctgtaggtatctcagttcggtgtaggtcgttcgctccaagctgggctgtgt
gcacgaaccccccgttcagcccgaccgctgcgccttatccggtaactatcgtcttgagtccaacccggtaagacacgactt
atcgccactggcagcagccactggtaacaggattagcagagcgaggtatgtaggcggtgctacagagttcttgaagtggt
ggcctaactacggctacactagaagaacagtatttggtatctgcgctctgctgaagccagttaccttcggaaaaagagttggt
agctcttgatccggcaaacaaaccaccgctggtagcggtggtttttttgtttgcaagcagcagattacgcgcagaaaaaaag
gatctcaagaagatcctttgatcttttctacggggtctgacgctcagtggaacgaaaactcacgttaagggattttggtcatga
gattatcaaaaaggatcttcacctagatccttttaaattaaaaatgaagttttaaatcaatctaaagtatatatgagtaaacttggt
ctgacagttaccaatgcttaatcagtgaggcacctatctcagcgatctgtctatttcgttcatccatagttgcctgactccccgtc
gtgtagataactacgatacgggagggcttaccatctggccccagtgctgcaatgataccgcgagacccacgctcaccggc
tccagatttatcagcaataaaccagccagccggaagggccgagcgcagaagtggtcctgcaactttatccgcctccatcca
gtctattaattgttgccgggaagctagagtaagtagttcgccagttaatagtttgcgcaacgttgttgccattgctacaggcatc
gtggtgtcacgctcgtcgtttggtatggcttcattcagctccggttcccaacgatcaaggcgagttacatgatcccccatgttg
tgcaaaaaagcggttagctccttcggtcctccgatcgttgtcagaagtaagttggccgcagtgttatcactcatggttatggca
gcactgcataattctcttactgtcatgccatccgtaagatgcttttctgtgactggtgagtactcaaccaagtcattctgagaata
gtgtatgcggcgaccgagttgctcttgcccggcgtcaatacgggataataccgcgccacatagcagaactttaaaagtgct
catcattggaaaacgttcttcggggcgaaaactctcaaggatcttaccgctgttgagatccagttcgatgtaacccactcgtg
cacccaactgatcttcagcatcttttactttcaccagcgtttctgggtgagcaaaaacaggaaggcaaaatgccgcaaaaaa
gggaataagggcgacacggaaatgttgaatactcatactcttcctttttcaatattattgaagcatttatcagggttattgtctcat
gagcggatacatatttgaatgtatttagaaaaataaacaaataggggttccgcgcacatttccccgaaaagtgccacctgac
gtctaagaaaccattattatcatgacattaacctataaaaataggcgtatcacgaggccctttcgtc

MS2 tag-Puro-polyA

Puromycin

LoxP
4XMS2
sgRNA
polyA
TCGCGCGTTTCGGTGATGACGGTGAAAACCTCTGACACATGCAGCTCCCG
GAGACGGTCACAGCTTGTCTGTAAGCGGATGCCGGGAGCAGACAAGCCC
GTCAGGGCGCGTCAGCGGGTGTTGGCGGGTGTCGGGGCTGGCTTAACTAT
GCGGCATCAGAGCAGATTGTACTGAGAGTGCACCATATGATAACTTCGTA
TAGCATACATTATACGAAGTTATTTTACCGGTCGGGAATTCGGCCAGTACC
CAAAAAGCGGGGGGCGCGCCGGGGTCTGGTGGCAGTGGAGGGGGGTCC
TACCCATACGATGTTCCAGATTACGCTTGATTACCCCTATGATGTGCCTGA
CTACGCATGATTACCCATACGATGTTCCAGATTACGCTGTCGACGGCCAA
CATGAGGATCACCCATGTCTGCAGGGCCTTTTTGGCCAACATGAGGATCA
CCCATGTCTGCAGGGCCTTTTTGGCCAACATGAGGATCACCCATGTCTGCA
GGGCCTTTTTGGCCAACATGAGGATCACCCATGTCTGCAGGGCCGCGCCA
TAACTTCGTATAGCATACATTATACGAAGTTATCGGGATCCCATGACCGA
GTACAAGCCCACGGTGCGCCTCGCCACCCGCGACGACGTCCCCAGGGCCG
TACGCACCCTCGCCGCCGCGTTCGCCGACTACCCCGCCACGCGCCACACC
GTCGATCCGGACCGCCACATCGAGCGGGTCACCGAGCTGCAAGAACTCTT
CCTCACGCGCGTCGGGCTCGACATCGGCAAGGTGTGGGTCGCGGACGACG
GCGCCGCCGTGGCGGTCTGGACCACGCCGGAGAGCGTCGAAGCGGGGGC
GGTGTTCGCCGAGATCGGCCCGCGCATGGCCGAGTTGAGCGGTTCCCGGC
TGGCCGCGCAGCAACAGATGGAAGGCCTCCTGGCGCCGCACCGGCCCAA
GGAGCCCGCGTGGTTCCTGGCCACCGTCGGAGTCTCGCCCGACCACCAGG
GCAAGGGTCTGGGCAGCGCCGTCGTGCTCCCCGGAGTGGAGGCGGCCGA
GCGCGCCGGGGTGCCCGCCTTCCTGGAAACCTCCGCGCCCCGCAACCTCC
CCTTCTACGAGCGGCTCGGCTTCACCGTCACCGCCGACGTCGAGGTGCCC
GAAGGACCGCGCACCTGGTGCATGACCCGCAAGCCCGGTGCCTGACAGAT
CTTAGCCGCGGCCGCACAACTTGTTTATTGCAGCTTATAATGGTTACAAAT
AAAGCAATAGCATCACAAATTTCACAAATAAAGCATTTTTTTCACTGCATT
CTAGTTGTGGTTTGTCCAAACTCATCAATGTATCTTAGCTCTTCCGCTTCCT
CGCTCACTGACTCGCTGCGCTCGGTCGTTCGGCTGCGGCGAGCGGTATCA
GCTCACTCAAAGGCGGTAATACGGTTATCCACAGAATCAGGGGATAACGC
AGGAAAGAACATGTGAGCAAAAGGCCAGCAAAAGGCCAGGAACCGTAAA
AAGGCCGCGTTGCTGGCGTTTTTCCATAGGCTCCGCCCCCCTGACGAGCAT
CACAAAAATCGACGCTCAAGTCAGAGGTGGCGAAACCCGACAGGACTAT
AAAGATACCAGGCGTTTCCCCCTGGAAGCTCCCTCGTGCGCTCTCCTGTTC
CGACCCTGCCGCTTACCGGATACCTGTCCGCCTTTCTCCCTTCGGGAAGCG
TGGCGCTTTCTCATAGCTCACGCTGTAGGTATCTCAGTTCGGTGTAGGTCG
TTCGCTCCAAGCTGGGCTGTGTGCACGAACCCCCCGTTCAGCCCGACCGCT
GCGCCTTATCCGGTAACTATCGTCTTGAGTCCAACCCGGTAAGACACGAC
TTATCGCCACTGGCAGCAGCCACTGGTAACAGGATTAGCAGAGCGAGGTA
TGTAGGCGGTGCTACAGAGTTCTTGAAGTGGTGGCCTAACTACGGCTACA
CTAGAAGAACAGTATTTGGTATCTGCGCTCTGCTGAAGCCAGTTACCTTCG

GAAAAAGAGTTGGTAGCTCTTGATCCGGCAAACAAACCACCGCTGGTAGC
GGTGGTTTTTTTGTTTGCAAGCAGCAGATTACGCGCAGAAAAAAAGGATC
TCAAGAAGATCCTTTGATCTTTTCTACGGGGTCTGACGCTCAGTGGAACGA
AAACTCACGTTAAGGGATTTTGGTCATGAGATTATCAAAAAGGATCTTCA
CCTAGATCCTTTTAAATTAAAAATGAAGTTTTAAATCAATCTAAAGTATAT
ATGAGTAAACTTGGTCTGACAGTTACCAATGCTTAATCAGTGAGGCACCT
ATCTCAGCGATCTGTCTATTTCGTTCATCCATAGTTGCCTGACTCCCCGTC
GTGTAGATAACTACGATACGGGAGGGCTTACCATCTGGCCCCAGTGCTGC
AATGATACCGCGAGACCCACGCTCACCGGCTCCAGATTTATCAGCAATAA
ACCAGCCAGCCGGAAGGGCCGAGCGCAGAAGTGGTCCTGCAACTTTATCC
GCCTCCATCCAGTCTATTAATTGTTGCCGGGAAGCTAGAGTAAGTAGTTCG
CCAGTTAATAGTTTGCGCAACGTTGTTGCCATTGCTACAGGCATCGTGGTG
TCACGCTCGTCGTTTGGTATGGCTTCATTCAGCTCCGGTTCCCAACGATCA
AGGCGAGTTACATGATCCCCCATGTTGTGCAAAAAAGCGGTTAGCTCCTT
CGGTCCTCCGATCGTTGTCAGAAGTAAGTTGGCCGCAGTGTTATCACTCAT
GGTTATGGCAGCACTGCATAATTCTCTTACTGTCATGCCATCCGTAAGATG
CTTTTCTGTGACTGGTGAGTACTCAACCAAGTCATTCTGAGAATAGTGTAT
GCGGCGACCGAGTTGCTCTTGCCCGGCGTCAATACGGGATAATACCGCGC
CACATAGCAGAACTTTAAAAGTGCTCATCATTGGAAAACGTTCTTCGGGG
CGAAAACTCTCAAGGATCTTACCGCTGTTGAGATCCAGTTCGATGTAACC
CACTCGTGCACCCAACTGATCTTCAGCATCTTTTACTTTCACCAGCGTTTCT
GGGTGAGCAAAAACAGGAAGGCAAAATGCCGCAAAAAAGGGAATAAGG
GCGACACGGAAATGTTGAATACTCATACTCTTCCTTTTTCAATATTATTGA
AGCATTTATCAGGGTTATTGTCTCATGAGCGGATACATATTTGAATGTATT
TAGAAAAATAAACAAATAGGGGTTCCGCGCACATTTCCCCGAAAAGTGCC
ACCTGACGTCTAAGAAACCATTATTATCATGACATTAACCTATAAAAATA
GGCGTATCACGAGGCCCTTTCGTC
